# Muscle-specific inositide phosphatase (MIP/MTMR14) is reduced with age and its loss accelerates skeletal muscle aging process by altering calcium homeostasis

**DOI:** 10.18632/aging.100190

**Published:** 2010-08-25

**Authors:** Sandra Romero-Suarez, Jinhua Shen, Leticia Brotto, Todd Hall, ChengLin Mo, Héctor H. Valdivia, Jon Andresen, Michael Wacker, Thomas M. Nosek, Cheng-Kui Qu, Marco Brotto

**Affiliations:** ^1^ Muscle Biology Research Group-MUBIG, Schools of Nursing, University of Missouri-Kansas City, Kansas City, MO 64108, USA; ^2^ Department of Medicine, School of Medicine, Case Western Reserve University, Cleveland, OH 44106, USA; ^3^ Department of Physiology, School of Medicine and Public Health, University of Wisconsin, Madison, WS 53711, USA; ^4^ School of Medicine, University of Missouri-Kansas City, Kansas City, MO 64108, USA; ^5^ Department of Physiology & Biophysics, School of Medicine, Case Western Reserve University, Cleveland, OH 44106, USA; ^6^ Muscle Biology Research Group-MUBIG, School of Biological Sciences, University of Missouri-Kansas City, Kansas City, MO 64108, USA; * These authors contributed equally to this work

**Keywords:** MIP/MTMR14, muscle aging, sarcopenia, skeletal muscle, intracellular calcium homeostasis

## Abstract

We have recently reported that a novel muscle-specific inositide phosphatase (MIP/MTMR14) plays a critical role in [Ca^2+^]_i_ homeostasis through dephosphorylation of sn-1-stearoyl-2-arachidonoyl phosphatidylinositol (3,5) bisphosphate (PI(3,5)P2). Loss of function mutations in MIP have been identified in human centronuclear myopathy. We developed a MIP knockout (MIPKO) animal model and found that MIPKO mice were more susceptible to exercise-induced muscle damage, a trademark of muscle functional changes in older subjects. We used wild-type (Wt) mice and MIPKO mice to elucidate the roles of MIP in muscle function during aging. We found MIP mRNA expression, MIP protein levels, and MIP phosphatase activity significantly decreased in old Wt mice. The mature MIPKO mice displayed phenotypes that closely resembled those seen in old Wt mice: i) decreased walking speed, ii) decreased treadmill activity, iii) decreased contractile force, and iv) decreased power generation, classical features of sarcopenia in rodents and humans. Defective Ca^2+^ homeostasis is also present in mature MIPKO and old Wt mice, suggesting a putative role of MIP in the decline of muscle function during aging. Our studies offer a new avenue for the investigation of MIP roles in skeletal muscle function and as a potential therapeutic target to treat aging sarcopenia.

## Introduction

Aging is a complex biological process marked by the gradual decline of a multitude of physiological processes/functions that ultimately results in death [1-5]. Normal aging results in sarcopenia, the decreased muscle mass and function that develops despite interventions such as increased physical activity and improved diet [6,7].  While these interventions have proven to be effective in ameliorating the loss of muscle function with age, there is no intervention that can completely prevent or reverse sarcopenia.

The decline in muscle function (force and power) that results from sarcopenia is a major cause of restricted activity, muscle injuries, and loss of independence in older individuals. As populations age and live longer, this problem will continue to grow. The world wide cost of managing the consequences of sarcopenia is astronomical estimated in the hundreds of billions of dollars. Research designed to reveal the cellular mechanisms that contribute to sarcopenia and other age-related muscle disorders is essential for the development of effective treatments that can improve health outcomes for older adults.

It has been shown that the decrease in force and power that functionally characterize sarcopenia cannot be completely explained by atrophy alone [4,8,9]. Some of the mechanisms suggested to explain the discrepancy between atrophy-dependent vs. atrophy-independent loss of muscle function in aging include decreased myosin force and/or actin-myosin cross-bridge stability [8,9] and defective excitation-contraction coupling (ECC) [4;10]. Our research groups have contributed to the field of muscle aging by demonstrating that specific aspects of the excitation-contraction coupling (ECC) process are compromised with age [11,12].

While aging is a multigene phenomenon [13-15], we have focused our most recent studies on a new protein, muscle-specific inositide phosphatase (MIP), also known as myotubularin-related protein 14 (MTMR14) [16]. In a recent report, we characterized its basic functions in skeletal muscle [16]. Our studies showed that MIP is important in the ECC process of skeletal muscle (particularly influencing store-operated calcium entry (SOCE), calcium (Ca^2+^) storage and Ca^2+ ^release from the sarcoplasmic reticulum (SR).

In the current study, we have used a combination of approaches to phenotypically compare mature mice lacking MIP (MIPKO) with old wild type (Wt) mice. We also measured the cellular expression, concentration, and activity of MIP within muscle fibers with age.  These findings were correlated with functional outcomes and revealed that key features of sarcopenia manifest in the MIPKO much earlier (12-14 months) than in wild-type mice (22-24 months). The significant decrease in MIP mRNA expression, MIP protein content and MIP activity in normal, old Wt mice along with the striking phenotypic similarities between mature MIPKO and old Wt mice, suggest a putative role of MIP in the aging decline in muscle function.

## Results

### In vivo studies of activity: young and mature MIPKO mice behave like old WT mice

 In our recently published study [16], we showed that in a rotarod function test, the latency of MIPKO mice to fall off the rotating rod was decreased. In the inclined screen test, the percentage of MIPKO mice that could reach to the top of the inclined screen was greatly decreased compared to that of Wt littermates. These findings are very similar to results obtained in old Wt mice. To broaden the phenotypic comparison between MIPKO and Wt mice, we used the force-plate actimeter measurements [17]. All mice tested remained in the actimeter for 40 min, and we found that young Wt mice (4-6 month, n=58) walked 280 ± 27 meters, mature Wt (12-14 month, n=20) walked 283 ± 23 meters, and old Wt (22-24 month, n=12) walked 175 ± 32 meters. In contrast, young MIPKO mice (4-6 month, n = 12) walked 240 ± 18 meters, mature MIPKO walked 200 ± 22 (12-14 month, n=12), and old MIPKO (18-20 month, n=12) walked 155 ± 13 meters. These studies show that mature MIPKO mice behave like old Wt mice with respect to levels of spontaneous physical activity.

### Treadmill stress test reveals additional similarities between the mature MIPKO and old Wt mice

These series of experiments were designed to test the effects of stress of running until exhaustion in a treadmill. Thus, untrained Wt and MIPKO mice were ran in a rodent treadmill until exhausted as previously described by our group [18]. The differences we found were dramatic. Young (n =12), mature (n = 18), and old Wt (n = 12) respectively ran 33 ± 5, 38 ± 3, and 13 ± 5 min. In contrast, young (n =12), mature (n = 18), and old MIPKO (n = 12) respectively ran 22 ± 4, 10 ± 3, and 8 ± 5 min. These results are very intriguing, particularly when we contrasted the average running time of ~10 min in mature MIPKO mice with the running time of old (22-24 month) Wt mice of 13 ± 5 min. These results strongly suggest that locomotor dysfunction has an earlier onset in MIPKO mice. Furthermore, it illustrates the dramatic similarities between mature MIPKO mice and old Wt littermates. We have also consistently observed that at this age, MIPKO mice are significantly less active in their cages as further substantiated by our actimeter studies.

### Accelerated muscle wasting in MIPKO mice might explain defective in vivo function and suggest earlier onset of sarcopenia

**Figure 1.**
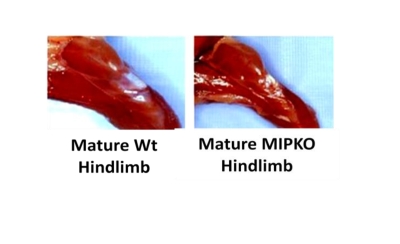
Muscle wasting develops prematurely in the MIPKO mice. Digital photography demonstrates the typical hindlimb size difference between 18-month old Wt and 18-month old MIPKO mice.

**Figure 2.**
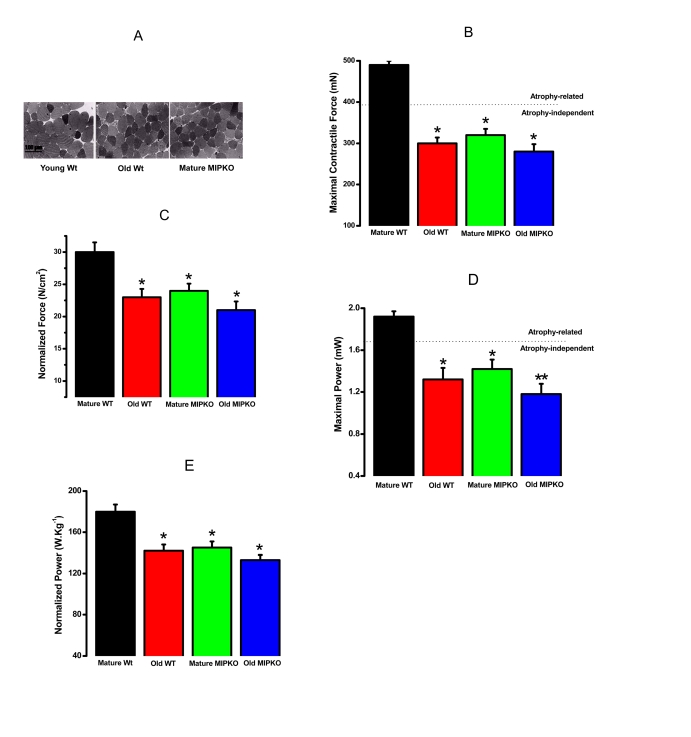
Evidence for muscle atrophy, decreased contractile force, and reduced power in skeletal muscles suggested similarity from old WT and MIPKO mice. In all figures, the black bars are mature, wild type mice, the red bars are old, wild type mice, the green bars are mature MIPKO mice, and the blue bars are old, MIPKO mice. (**A**) Typical Toluidine blue-stained cross sections of EDL muscles from young Wt, old Wt, and mature MIPKO mice. The cross-sectional areas of old Wt and MIPKO cells are significantly reduced compared with those of the young Wt. (**B**) Maximal contractile force in EDL muscle for each genotype. Atrophy (decrease in muscle cross-sectional area) can explain ~ 1/2 of the drop in total force (note the dotted horizontal line), but does not account for the complete decrease in contractile force. (**C**) Data from **B**, except that force is normalized per cross-sectional area (N/cm2). This figure illustrates the atrophy-independent component of contractile dysfunction. (**D**) Maximal power in EDL muscle from all four animal models. (**E**) Data from panel **D** was normalized per cross sectional area of muscles. It shows that a significant drop in power is atrophy-independent. Data is the average ± SE of 24 EDL muscles from 12 mice for each genotype. * indicates a significant difference (p < 0.01) between the control muscles and a particular genotype. ** indicates a significant difference (p < 0.01) between the old MIPKO mice and the old Wt and mature MIPKO mice.

We previously demonstrated that an 18-month old MIP KO mouse has ~40-50% less body mass as compared to the Wt littermates (See Figure 2 of our recent publication [16]). As seen in Figure 1, we now show that hindlimb muscle mass is also significantly reduced in the MIPKO mice when compared with Wt littermates, suggesting premature development of sarcopenia when MIP is ablated. These data might also help in explaining the reduced physical vigor encountered in MIPKO mice.

### Contractile force and power generation are reduced in skeletal muscles from mature MIPKO in a fashion similar to that of old Wt mouse

A functional corollary of sarcopenia in humans is the presence of decreased force and power. Figure 2A illustrates that the cross sectional area of skeletal muscle fibers from old Wt and mature MIPKO mice is reduced compared with mature Wt mice.  Figures 2B and 2D illustrate that the maximal contractile force and power in old Wt, mature MIPKO, and old MIPKO mice are significantly reduced from values found in mature Wt mice.  Atrophy can account for a reduction in force and power of approximately 25% (the dotted line in Figures 2B and2D) in the old and MIPKO animals.  When maximal force and power are normalized to cross sectional area (Figures 2C and 2E), it is clear that there is a reduction in force and power that are independent of muscle wasting and result from changes in the contractile properties of the muscle fibers. Strikingly, the same functional changes that manifest at 24 months in muscles from old Wt mice manifest by 12 months of age in MIPKO mice, suggesting that MIP is an important modulator of contractile function during aging.

### Downregulation of MIP expression and function with normal muscle aging

**Figure 3.**
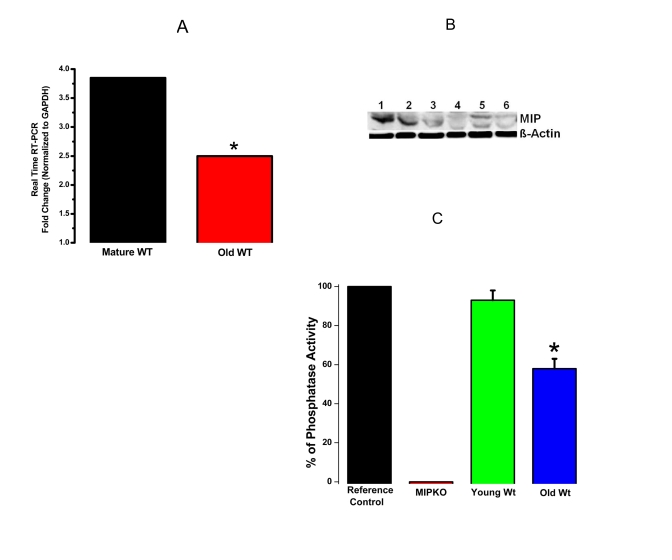
Reduced MIP gene expression, MIP protein levels and MIP phosphatase activity in old skeletal muscles. (**A**) Significant reduction in MIP expression in EDL muscles from old Wt mice (red bar) compared with mature Wt mice (black bar). (**B**) MIP protein content decreased drastically in old skeletal muscle. Lanes 1-3, mature Wt EDL; Lanes 4-6, old Wt EDL; β-actin as controls. (**C**) MIP enzymatic phosphatase activity reduced by ~ 30% in old Wt EDL muscles as compared to young Wt EDL muscles. * indicates a significant difference (p < 0.01) between the control muscles and a particular genotype.

Figure 3 shows down-regulation of MIP in Wt mice during aging using 3 different approaches (MIP gene expression, MIP protein content, and MIP phosphatase activity). These data show that MIP is tightly regulated at all levels, from transcription to translation to activity levels, suggesting an important physiological role for this muscle phosphatase. These data also provide strong evidence for the involvement of MIP in the development of sarcopenia.

These new data in Figure 3 not only address a specific concern from the review panel, but are equally exciting for the future of our proposed studies and the field of sarcopenia.

### Dysfunctional Ca^2+^ homeostasis in old WT and mature MIPKO

**Figure 4.**
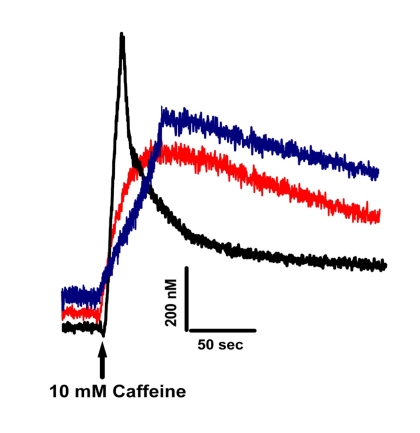
** Altered Ca^+2^ homeostasis is present in muscle fibers from old Wt and MIPKO mice.** Original traces representative of caffeine-induced Fura-2 Ca^+2^ transients in mature Wt (black trace), old Wt (red trace), and mature MIPKO FDB muscle fibers (blue trace). Examples shown are representative of 6-12 muscle fibers from 3 mice, and data were normalized to the intracellular Ca^+2^ concentrations in nM.

**Figure 5.**
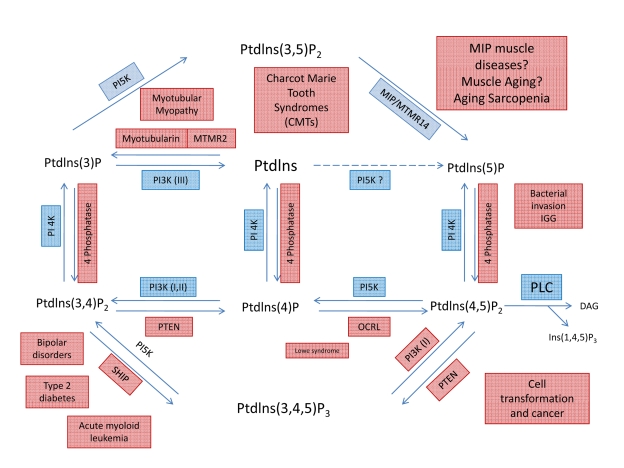
Metabolic pathways showing the interconversion of PIPs and putative roles of MIP and PI(3,5)P2. Through the specific action of different phosphatases, seven phosphoinosites molecules are generated. Note that a loss of MIP activity will lead to an increase in PI(3,5)P2 concentration and activity. Even small changes in content or activity of phosphatases and/or their substrates can lead to complex pathologies, including several muscle diseases.

Figure 4 shows that Ca^2+^ homeostasis is disrupted in both old Wt and mature MIPKO FDB muscle fibers. Three key features of intracellular Ca^2+^ homeostasis were similarly affected by old age and MIP ablation. First, resting levels of Ca^2+^ were higher in old Wt and MIPKO muscle fibers. Second, SR Ca^2+^ release triggered by caffeine was reduced in muscle fibers from both old Wt and mature MIPKO mice. Third, while the Ca^2+^ transient has a fast relaxation recovery in young Wt, it is significantly delayed in old Wt and MIPKO. We previously showed that intracellular elevation of PI(3,5)P2 due to the absence of MIP (see Figure 5) led to chronic activation of the ryanodine receptor (RyR1) and inhibition of store-operated Ca^2+^ entry (SOCE) [16], which supports the present findings of altered Ca^2+^ homeostasis with age or in the MIPKO subjects when MIP is downregulated and therefore PI(3,5)P2 is elevated. Undoubtedly, with chronic activation of the RyR1 by PI(3,5)P2, one predicts that resting levels of Ca^2+^ will be higher and Ca^2+^ storage and Ca^2+ ^transients reduced. This combination of effects is certainly detrimental to skeletal muscle function and will contribute to the decline in muscle strength with aging.

## Discussion

This communication and our previous study [16] elevate the role of MIP as a potent regulator of skeletal muscle function under normal conditions. The fact that MIP expression, concentration, and function are decreased with age also shows that MIP is important for, or at least contributes to, the development of sarcopenia. Furthermore, we have demonstrated that PI(3,5)P2 is the major substrate for MIP and that reduction in MIP levels leads to accumulation of PI(3,5)P2 within the membrane of the muscle SR.

Thus, the significant decrease in MIP phosphatase activity during aging should induce accumulation of intracellular PI(3,5)P2, leading to Ca^2+^ homeostasis defects, which is precisely the phenotype identified in both old Wt and mature MIPKO muscle fibers.

Phospholipids and phosphoinosites were once thought to play only structural and energetic functions. They are now recognized as signaling molecules,  particularly  as second messengers [19-26]. PIPI(3,5)P2, discovered only 10 years ago, is an isomer of the well-characterized phosphoinosite, PI(3,5)P2. PI(3,5)P2 has been extensively studied and found to modulate the properties of many membrane channels. The seven known PIPs (see Figure 5) are thought to form complex signal transduction networks in organisms spanning yeasts to humans. The broadness of cellular functions controlled and modulated by lipids seems almost unlimited with defined roles in cell signal transduction, proliferation, growth, apoptosis, immune response, and adaptation to stress, modulation of ionic channels and cell transporters [27].

Each PIP binds to a distinctive set of effector proteins and, thereby, regulates a characteristic suite of cellular processes, including membrane trafficking, cell survival/growth, cell division, and cellular motility [28] (See also Figure 5). Importantly, aberrant lipid metabolism often leads to the onset of pathology, and thus the precise balance of signaling lipids and their effectors can serve as biomarkers for health and disease [19,22,27]. Therefore, the increased intracellular levels of PI(3,5)P2 that result from either MIP ablation or from the natural decrease in MIP function with aging might induce significant imbalances in signaling pathways.

The potent signaling properties of PIPs depend on their localization as well as abundance, which are determined by the collective actions of PIP kinases, PIP phosphatases, and phospholipases [29,30]. PIP phosphatases (such as MIP) are a subfamily of phosphatases that hydrolyze PIPs [31] (Figure 5). For example, PTEN phosphatase dephosphorylates PI(3,4,5)P3 on the plasma membrane and is critical for a variety of cellular processes [32].  Loss of PTEN function is associated with the tumorigenesis of many types of cancers [32]. The role of other PIP phosphatases, such as myotubularin- and myopathy- related phosphatases (MTMR) have not been well characterized.  PIPs are also involved in bipolar disorder, myopathies, acute myeloid leukemia, and type-2 diabetes. Importantly, loss-of-function mutations in several MTMR phosphatases (MTM1, MTMR2, and MTMR13) have been identified in genetic conditions, namely X-linked myotubular myopathy (a muscle degenerative disease that shares some similarities with sarcopenia) and Type-4B Charcot-Marie-Tooth disease (a neurodegenerative condition) [33-36]. However, the molecular mechanisms by which the mutations in these phosphatases induce such diseases remain largely unknown [19,24].  As shown in Figure 5, the quick interconversion of seven different PIPs creates a dynamic signaling network that might underlie molecular mechanisms relevant for a myriad of diseases.

We believe that aging must be seen as the result of a multitude of long-term, cumulative adaptations and not only due to changes resulting from the ablation or the downregulation of a single gene. Nevertheless, changes in MIP function could cause a cascade of effects with consequences that could be far more serious and broader than the specific change in MIP itself. The reason for such assertion is rather simple. MIP controls the intracellular levels of PI[3,5]P2, which in turn binds to a multitude of proteins [28-30], therefore working as a principal molecule in the coordination of intracellular networks [28,31]. Intriguingly, we have recently obtained preliminary evidence that indicates that mature MIPKO develops cardiovascular diseases and osteoporosis, both in agreement of a broader and age-related role of MIP (Wacker, Andresen, Bonewald, Johnson & Brotto; unpublished observations). Furthermore, we previously showed that intracellular elevation of PI(3,5)P2 is able to activate the opening of the RyR1 at contracting or resting levels of Ca^2+^. Such an effect can lead to a vast amount of secondary changes as Ca^2+ ^itself is a second messenger that controls cellular life and death. For example, chronically elevated resting levels of intracellular Ca^2+^ as observed in mature MIPKO and old Wt muscle cells, might activate proteolytic enzymes or enhance the production of reactive oxygen species and free radicals, which in turn may contribute to muscle wasting.

In summary, our studies suggest new roles of MIP and its major substrate, PI(3,5)P2, in the decline in muscle function during aging. We believe that the loss of MIP activity with age could contribute to the loss of muscle function due to the buildup of PI(3,5)P2 within the muscle SR membrane, and consequently the increased conductance of the ryanodine receptor channel, loss of Ca^2+^ from the SR, and also inhibition of SOCE into the cell [16]. While additional studies will be required to better define the cell biological functions of MIP and PI(3,5)P2, we propose that the MIP-PI(3,5)P2 signaling pathway is an important contributor for aging sarcopenia, and as such could be explored as a new therapeutic option for the treatment of not only muscle myopathies, but also for aging sarcopenia. In addition, the MIPKO model seems suitable for understanding of some aspects of muscle aging and sarcopenia.

## Experimental procedures

Force Plate Actimeter (FPA). To complement our *in vivo* muscle function approaches we have selected the FPA because it provides an efficient, accurate, and highly reproducible means of assessing many aspects of locomotor behavior [17].

Ex-vivo, isolated muscle contractility protocols. These studies followed protocols established by Brotto & Nosek [37-39]. Intact extensor digitorum longus (EDL) muscle of male and female WT and MIPKO mice of all age groups were removed from tendon to tendon and immediately placed in a dissecting dish containing a modified bicarbonate Ringer solution with 2.5 mM extracellular Ca^2+^ or with 0 mM Ca plus 0.1 mM EGTA to test the effects of extracellular Ca^2+^ and SOCE in muscle function. The pH was adjusted to 7.4 with NaHCO_3_, followed by the addition of fetal bovine serum (to 0.2%) to increase viability of the dissected muscle [37,40].The solution was continuously aerated with a gas-mixture consisting of 95% O_2 _and5% CO_2_. EDL and SOL muscles were mounted vertically between two Radnoti (Monrovea, CA, USA) stimulating platinum electrodes and immersed in a 20 ml bathing chamber containing the incubation medium. Via the tendons, the muscles were suspended from movable isometric force transducers above the chambers and secured to the base of the tissue support within the chambers.  The analog output of the force transducer were digitized, stored and analyzed with PowerLab Software (Colorado Springs, CO, USA).  For each muscle, the resting tension and the stimulatory voltage was provided by a Grass S8800 digital stimulator (West Warwick, RI, USA) and adjusted to produce a maximal isometric tetanic force (T_max_). EDL muscles were the muscle choice since effects of Sarcopenia are known to be exacerbated in fast-twitch muscles such as the EDL. *Equilibration*: The intact muscles were allowed a 20-minute equilibration period after which time they were stimulated with pairs of alternating high (that produced T_max_) and low (that produced 1/2 T_max_) frequency pulse-trains administered with a periodicity of 1 minute.  Utilization of the proposed paradigm of stimulation helps with the study of the relative contributions of the contractile proteins (T_max_) and the SR (1/2 T_max_) to contractile function [37].

Force vs. frequency relationship. Following equilibration, the muscles are subjected to stimulation with frequencies ranging from 1-200 Hz to generate the force vs. frequency (FF) relationship. *Force Normalization:* All force data are normalized as either the absolute force (force per cross sectional area) or as a percentage of the maximum tetanic force (%T_max_) measured before the beginning of the fatiguing protocol.

Fura-2 monitoring of intracellular Ca^2+^. For quantitative measurements of intracellular [Ca^2+^], flexor digitorum brevis (FDB) musclefibers were utilized. FDB muscle fibers were enzymatically isolated in a 0 Ca Tyrode solution containing 2 mg/mL type I collagenase for 2 hours in a shaking bath at 37°C. FDB muscle fibers were then transferred to a 0 Ca^2+^ Tyrode solution without collagenase and gently triturated with a pipette.  The fibers were then be loaded with 5 μM Fura-2-AM for 40 minutes, after which the Fura-2 AM was washed off and be allowed to de-esterify. As fiber motion artifacts are associated with intracellular Ca^2+ ^release, 20 μM N-benzyl-p-toluene sulfonamide (Sigma), a specific myosin II inhibitor, was then applied for 20 minutes.  A dual-wavelength(excitation at 340 nm and 380 nm) PTI spectrofluorometer (PhotonTechnology International, Birmingham, NJ) was used to determinethe magnitude and kinetic changes of caffeine-induced intracellular Ca^2+^ transients. Ratiometric changes were converted into relative levels of Ca^2+^ in nM as previously detailed by Brotto et al [41].

Biochemical profiling of skeletal muscles. Freshly isolated EDL muscles were dissected from all mice and saved for biochemical analyses. Gene expression, MIP protein content, and MIP activity assays were performed on these muscles as previously described by Zhao et al. [11] and recently modified by Shen et al [16].

MIP gene expression and MIP protein expression. These procedures were performed as described in Zhao et al, and as recently modified in Shen et al [16]. Briefly, the mRNA expression level of MIP gene was determined by qPCR in freshly isolated EDL muscles after mRNA was extracted using a RNeasy mini kit (Qiagen, Valencia, CA, USA) and transcribed into cDNA by M-MLV Reverse Transcriptase (Promega, Madison, WI, USA).  qPCR was performed using SYBR Green PCR supermix (Invitrogen, Carlsbad, CA, USA) on a Bio-Rad MyIQ 96-well PCR detection system. The glyseraldehyde-3-phosphate dehydrogenase (GAPDH) gene was used as the reference gene. Quality of the amplicons was confirmed by detection of uniform melting curve peaks for each gene. One hundred nanograms of cDNA were added per reaction and the final primer concentration was 200 nM. Experiments were run in triplicate. Relative Ct values were calculated as 2^CtGAPDH-CtTarget^. Protein concentrations were determined by DC protein assay (Bio-Rad) and 10 μg per sample was separated by SDS-polyacrylamide gel electrophoresis at room temperature on 4-12% Tris-glycine gradient gels for 2h at 60 mAmps on a Mini PROTEAN II gel system (Bio-Rad). Gels were loaded in parallel and one set was stained with Novex Colloidal Blue stain (Invitrogen), per manufacturer's instructions. Equivalent loading was confirmed using monoclonal β-actin antibody (Sigma), 0.2 μg mL−1. Results were visualized with an ECL + kit (GE Healthcare, Piscataway, NJ, USA) following the manufacturer's directions.

MIP phosphatase activity. To determine the lipid phosphatase activity, Di-C_8_ phosphoinositides (Echelon Biosciences Inc., Salt Lake City, UT) and dioleoyl-phosphatidylserine (Sigma, St. Louis, MO) were resuspended via sonication in the assay buffer (100 mM sodium acetate, 50 mM bis-Tris, 50 mM Tris pH 5.5, and 10 mM dithiothreitol) to final concentrations of 100 and 1000 μM, respectively. Equal volumes of di-C_8_ phosphoinositides and dioleoyl-phosphatidylserine were added into 1.5 ml microcentrifuge tubes and the mixtures were prewarmed at 37°C for 5 min. Reactions were initiated by the addition of 500 ng of GST-MIP fusion protein diluted in the assay buffer containing 1.0 mg/ml gelatin. This reaction step provided the control values seen in Figure 4. Next, muscle homogenate reactions were initiated by the addition of 2000 ng of total muscle protein diluted in the assay buffer containing 1.0 mg/ml gelatin. Reactions were quenched after 30 min by the addition of 20 μl of 0.1 M *N*-ethylmaleimide and spun at 18,000 × g for 10 min to sediment the lipid aggregates.  The supernatant (25 μl) was added to a 96-well plate and 100 μl of Malachite green reagent (Echelon Biosciences Inc., Salt Lake City, UT) was added to each well.  After incubation at room temperature for 15 min, the color development was measured at 620 nm.  Inorganic phosphate release was quantitated using a standard curve generated with KH_2_PO_4_ in distilled H_2_O.

Statistical analyses. Values are mean ± SEM. Significance was determined by ANOVA followed by either Tukey's or Bonferroni's tests. ANOVA on Ranks followed by Kruskal-Wallis test was used for non-parametric data. A value of *P* < 0.05 was used as criterion for statistical significance.
